# Gene expression profile suggesting immunological dysregulation in two Brazilian Bloom's syndrome cases

**DOI:** 10.1002/mgg3.1133

**Published:** 2020-02-19

**Authors:** Marilia M. Montenegro, Caio R. Quaio, Patricia Palmeira, Yanca Gasparini, Andreia Rangel‐Santos, Julian Damasceno, Estela M. Novak, Thamires M. Gimenez, Guilherme L. Yamamoto, Rachel S. Ronjo, Gil M. Novo‐Filho, Samar N. Chehimi, Evelin A. Zanardo, Alexandre T. Dias, Amom M. Nascimento, Thais V. M. M. Costa, Alberto J. da S. Duarte, Luiz L. Coutinho, Chong A. Kim, Leslie D. Kulikowski

**Affiliations:** ^1^ Laboratorio de Citogenomica Departamento de Patologia Faculdade de Medicina FMUSP Universidade de Sao Paulo Sao Paulo SP Brazil; ^2^ Unidade de Genetica Departamento de Pediatria Instituto da Crianca, Hospital das Clinicas HCFMUSP Faculdade de Medicina Universidade de Sao Paulo Sao Paulo SP Brazil; ^3^ Laboratório de Pediatria Clínica Departamento de Pediatria Instituto da Crianca, Hospital das Clinicas HCFMUSP Faculdade de Medicina Universidade de Sao Paulo Sao Paulo SP Brazil; ^4^ Fundação Pró‐Sangue Hemocentro de São Paulo Sao Paulo SP Brazil; ^5^ Laboratório de Pesquisa Translacional em Oncohematologia Instituto de Tratamento de Cancer Infantil (ITACI) Sao Paulo SP Brazil; ^6^ Centro de Genomica Funcional, Departamento de Zootecnia, Universidade de São Paulo, Escola Superior de Agricultura Luiz de Queiroz ESALQ‐USP Piracicaba Brazil

**Keywords:** *BLM* gene, Transcriptome, Bloom's syndrome, Immunology, RNA‐Seq

## Abstract

**Background:**

Bloom syndrome (BS) is a rare autosomal recessive chromosome instability disorder. The main clinical manifestations are growth deficiency, telangiectasic facial erythema, immunodeficiency, and increased risk to develop neoplasias at early age. Cytogenetic test for sister chromatid exchanges (SCEs) is used as a diagnostic marker for BS. In addition, most patients also present mutations in the *BLM* gene, related to defects in the DNA repair mechanism. However, the molecular mechanism behind the pathogenicity of BS is still not completely understood.

**Methods:**

We describe two patients confirmed with BS by SCE and molecular analysis. Also, we performed the gene expression profile by the RNA‐seq methodology in mRNA transcripts for differential gene expression analysis using as a biological condition for comparison BS versus health controls.

**Results:**

We detected 216 differentially expressed genes related to immunological pathways such as positive regulation and activation of B cells, immune effector process and absence of difference of DNA repair genes expression. In addition; we also observed differentially expressed genes associated with apoptosis control, such as *BCL2L1*, *CASP7*, *CDKN1A*, *E2F2*, *ITPR, CD274*, *TNFAIP6*, *TNFRSF25, TNFRSF13C,* and *TNFRSF*17.

**Conclusion:**

Our results suggest that the combination of altered expression of genes involved in signaling pathways of immune response and apoptosis control may contribute directly to the main characteristics observed in BS, such as recurrent infections, growth failure, and high risk of cancer. Transcriptome studies of other instability syndromes could allow a more accurate analysis of the relevant gene interactions associated with the destabilization of the genome. This is a first description of the profile of differential gene expression related to immunological aspects detected in patients with BS by RNA‐seq.

## Graphical abstract

We investigate two patients with Bloom´s syndrome that presented immunological deficiency that was confirm by differential gene expression analysis by RNA‐seq using Illumina platform. The analysis showed an unexpected highly complex transcriptional profile with downregulated differentially expressed genes associated to activation and regulation of immune pathways and absence of difference of DNA repair genes. This is a first description of the profile of differential gene expression related to immunological aspects detected in patients with SB by RNA‐seq.

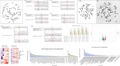

## 1 INTRODUCTION

Bloom's syndrome (BS; Online Mendelian Inheritance in Man database #21900) is a rare autosomal recessive chromosomal instability disorder characterized by severe growth deficiency (pre‐ and postnatal), sun‐sensitive facial erythema, immunological deficiency, and a remarkably increased risk of developing neoplasias of various types at a younger age than expected in the general population. The neoplasias are the main cause of death among affected individuals (Bloom, 1954; Chakraverty & Hickson, 1999; Brosh et al., 2001; Beamish et al., 2002; Bhisitkul & Rizen, 2004; Flanagan and Cunniff, 2006; Moreira et al., 2013).

The cells from patients display genomic instability characterized by increased frequency of chromosomal and chromatid gaps, breaks, and several chromosomal aberrations, especially quadriradial configurations and increased sister chromatid exchanges (SCEs) which constitute the hallmark of BS (Cunniff, Bassetti, & Ellis, 2017; Ellis, 1995). Chromosomal alterations observed in BS cells may generate repositioning of tumor suppressor genes that are important for regulation of development and function of T and B cells, thus predisposing to immunodeficiency and cancer development (Derks, Hoeijmakers, & Pothof, 2014; Ellis et al., 1998). Indeed, a recent immunological study of BS patients demonstrated clinically relevant increased number of infections, relatively low serum immunoglobulin, normal range of T, B, and NK cells and high percentage of CD4^+^ and CD8^+^ effector memory T cells (Schoenaker et al., 2018).

BS is associated with biallelic pathogenic mutations in the *BLM* gene, located in 15q26.1 region. In normal conditions, *BLM* encodes a 1,417 amino acids protein. It is member of the RecQ family with DNA helicase activity called BLM helicase or RECQL3 (Elmore, 2007; German, 1969a, 1993). BLM helicase is a nucleolar protein essential for the integrity and stability of DNA (German, 1997; German & Passarge, 1989). It is considerate anti‐recombinant key, because it is a DNA repair protein able to resolve DNA structures, like a Holliday junction, D‐loops, and G‐quadruplex generated during HRR caused by a DSB (Ellis et al., 1998; Hickson et al., 2001).

Cytogenetic study, previously considered the gold standard diagnostic tool for BS (Ellis et al., 1998), has been substituted for more accurate molecular analysis of *BML* gene. Recent technological advances with next‐generation sequencing made possible the rise of methodologies based on RNA‐Seq, which allows transcriptome studies, analysis of previously unidentified genes and splice variants, genomic variations, and a comprehensive expression profile. Indeed, the characterization of gene expression in cells via measurement of mRNA levels is a useful tool in determining how the transcriptional machinery of the cell is affected by external signals, genetic diseases or how cells differ between a healthy state and a disease state (Huang, Sherman, & Lempicki, 2009). The definition of an accurate map of all genes, with its alternative isoforms, could be critical for biological understanding of the disease. In this sense, we used RNA‐Seq to genomic instability in two patients affected by a BS.

## 2 PATIENTS AND METHODS

### 2.1 Patients

We compared gene expression patterns using an assay based on RNA‐Seq of mRNA transcriptome between two groups: BS patients versus healthy controls. BS patients group included two patients with definite diagnosis of BS previously confirmed by cytogenetic and molecular study of *BLM* gene, while healthy controls comprised three individuals (two males and one female) from general population. A summary of main findings of all samples is described in Table 1.

**Table 1:** Description of samples that compounded this work

Samples	DH	Gender	Age (years)
K1	BS	F	28
M1	BS	F	13
C1	H	M	29
C2	H	F	29
C3	H	M	31

Abbreviations BS, Bloom´s syndrome; C, Controls; DH, Diagnostic hypothesis; F: Female; H, Healthy; M: Male

The study was approved by ethics committee of the University of São Paulo HCFMUSP‐CAPPesq 63954/12 and all patients or their legal guardians signed a consent form.

**Figure 1:**
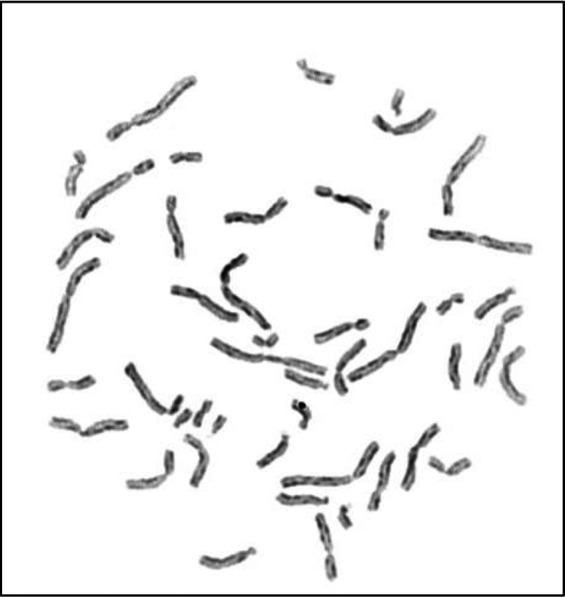
Peripheral blood lymphocyte metaphase of Patitent K1 with BrDU‐Hoeschst preparation demonstrating an increase of SCE

**Figure 2:**
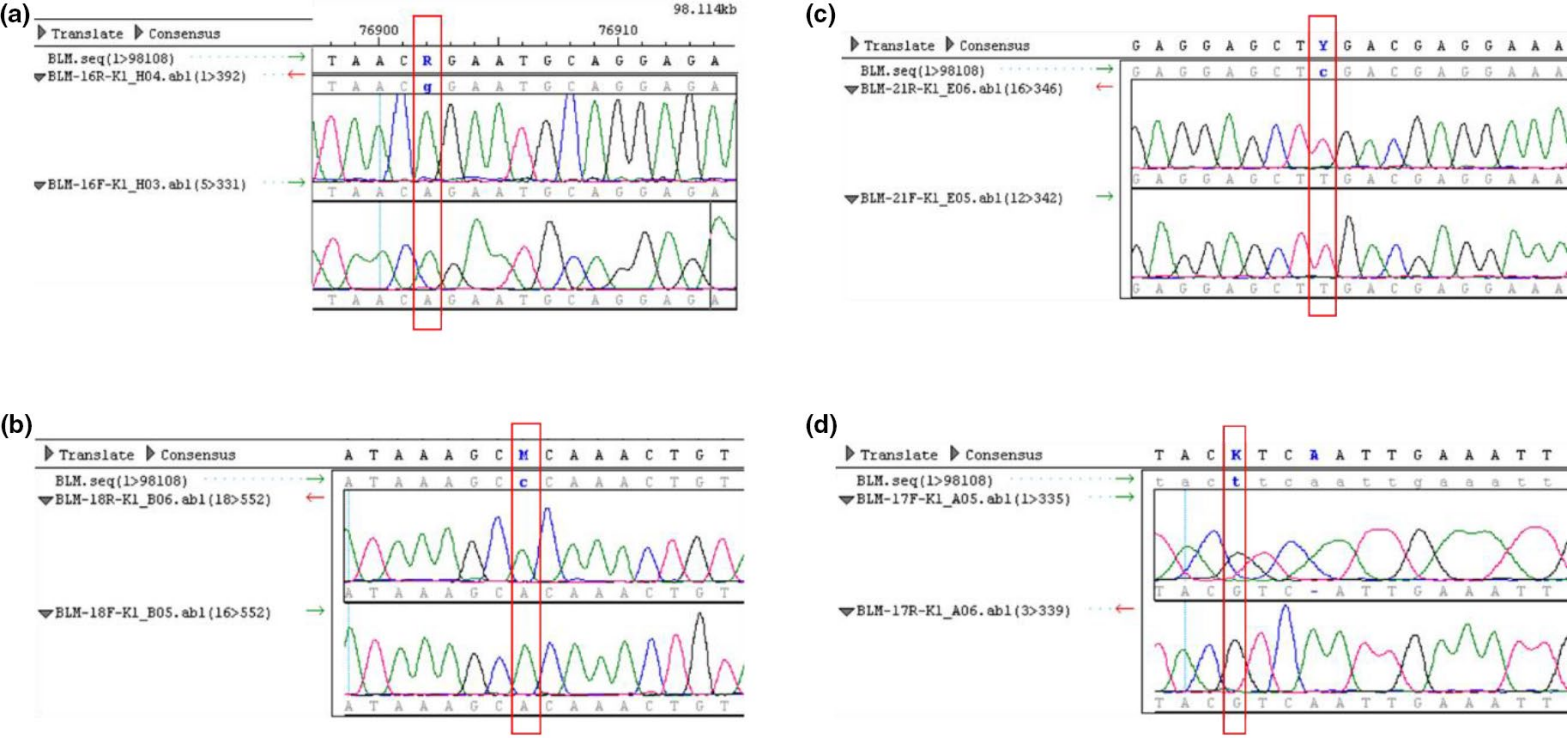
Electropherogram of Sanger analysis of Patient K1 demonstrating: (a) change of one Adenin (A) for Guanin (G) at the position 3102 of exon 16 of the *BLM* gene (c.3102G>A:p.Thr1034=); (b) change of one Citosin (c) for Adenin (A) at the position 3531 of exon 18 of the *BLM* gene (c.3531C>A:p.Ala1177=); (c) change of one Citosin (C) for Timin (T) at the position 3945 of exon 21 of the *BLM* gene (c.3945C>T:p.Leu1315=); (d) change of one Timin (T) for Guanin (G) at the position 3358 in intron between exons 17 and 18 of the *BLM* gene (c.3358+32T>G), observed in Patient K1

Patient K1 is a 28‐year‐old Brazilian woman, fifth child of consanguineous parents of non‐Jewish family that was born at 35 weeks of gestation with low birth weight (1800 g) and small length (40 cm) after intrauterine growth retardation. She had normal cognitive development, remarkable failure to thrive and recurrent infections (otitis, diarrhea, respiratory tract). Physical examination revealed microcephaly, facial dimorphisms (prominent nose, prominent ears, and malar hypoplasia), disseminated hyper and hypochromic macules, and decreased anthropometric measurements. Her immunological evaluation (Data S1: Supplementary Immunological Table K1 Patient) showed no remarkable alterations. The diagnosis of BS was established with cytogenetic study of sister chromatid exchange (SCE): karyotype of peripheral blood revealed increased frequency of SCE (60,9 SCE per metaphase) and chromosomal breakages (Figure 1). Molecular analysis of the *BLM* gene identified the presence of three homozygous synonymous variants and an intronic variant already described in dbSNP: c.3102G>A:p.Thr1034=, c.3531C>A:p.Ala1177=, and c.3945C>T:P.Leu1315=, depicted in Figure 2a,b,c,d. All variants were classified as benign according to ACMG guidelines.

**Figure 3:**
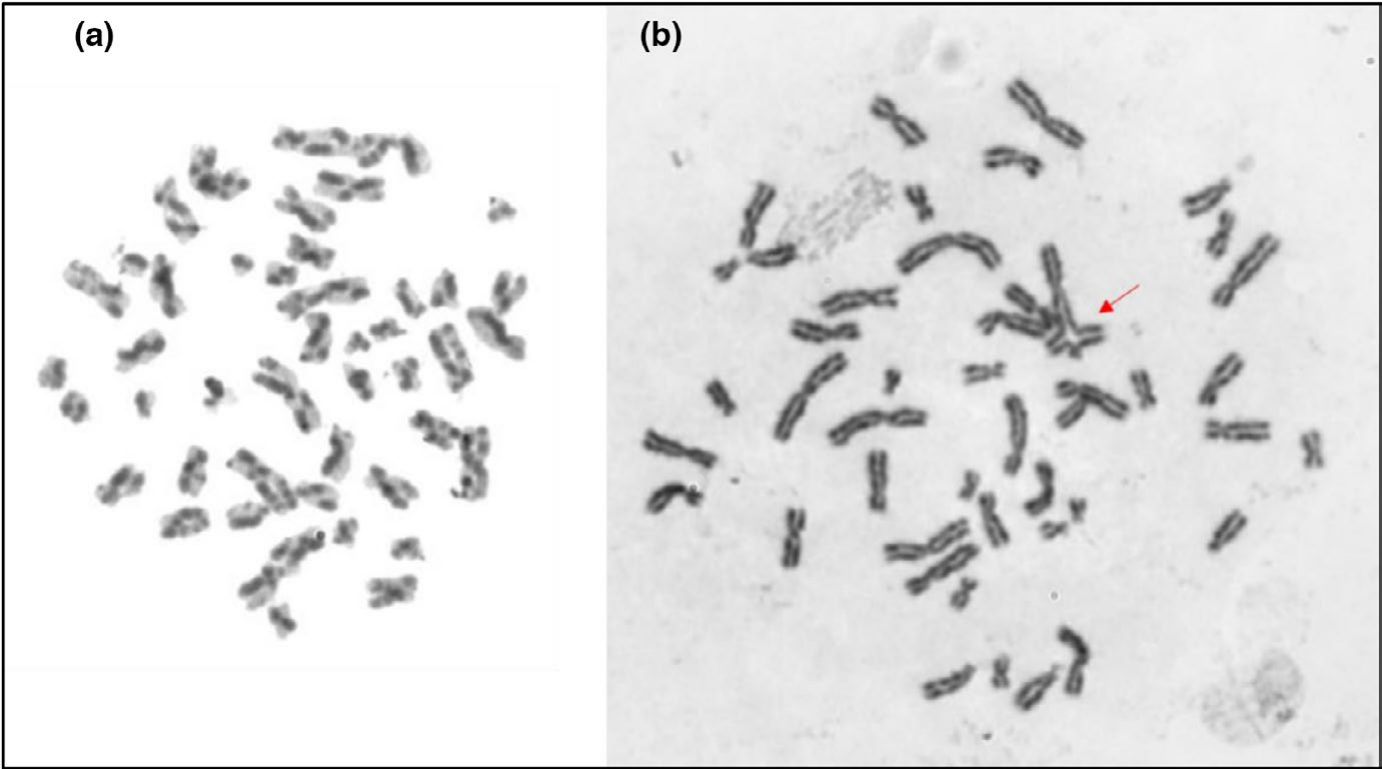
Peripheral blood lymphocyte metaphase of Patient M1 with BrDU‐Hoeschst preparation demonstrating an increase of sister chromatid exchange (a); and a very rare quadrirradial configuration (b)

**Figure 4:**
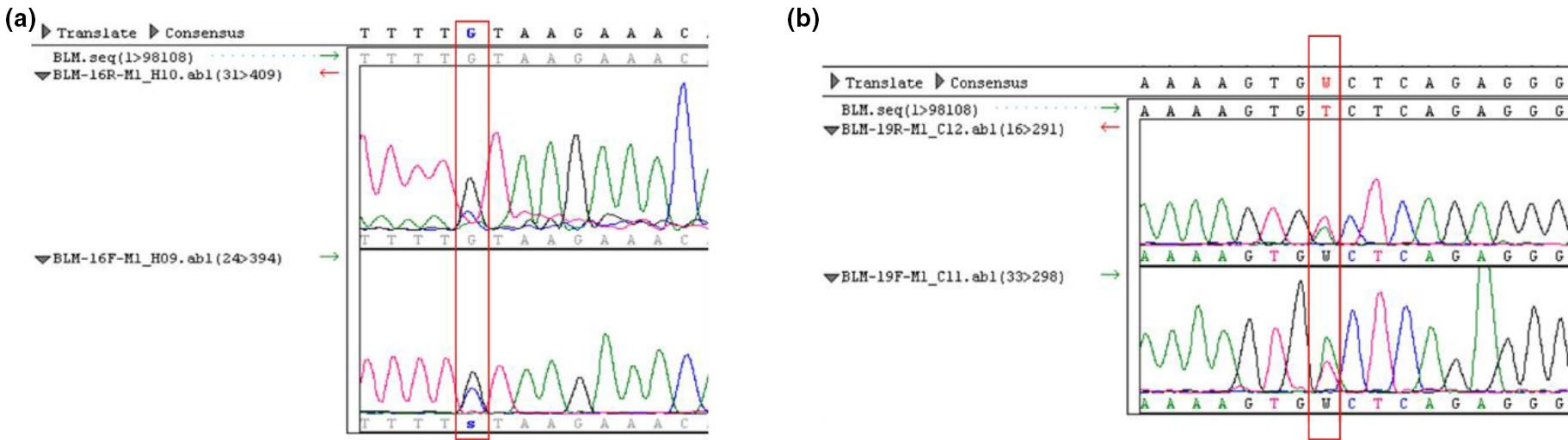
Electropherogram of Sanger analysis of Patient M1 demonstrating: (a) missense variation at the position 3164 of exon 16 of the *BLM* gene (c.3164G>C:p.Cys1055Ser) and (b) missense variation at the position 3625 of exon 19 of the *BLM* gene (c.3625T>A:p.Ser1209Thr)

Patient M1 is a 12‐year‐old Brazilian girl, the second child of non‐Jewish, non‐consanguineous parents. She was born at 28 weeks of high‐risk gestation with hemorrhage and loss of amniotic fluid with low birth weight (870 g) and small length (33 cm). She had learning disabilities, severe failure to thrive and recurrent infections (pneumonia, bronchitis). Her physical examination revealed characteristic features of BS, including microcephaly, facial dysmorphisms (prominent nose, prominent ears, and malar hypoplasia), hyper‐ and hypochromic macules, clinodactyly, and diminished anthropometric measurements. Her immunological evaluation (Data S1: Supplementary Immunological Table M1 Patient) showed no remarkable alterations. Cytogenetic analysis of peripheral blood revealed increased frequency of SCE (61,3 SCE per metaphase) and a very rare quadriarradial chromosome configuration (Figure 3). Molecular analysis showed two missense heterozygous variants already described in dbSNP: c.3164G>C:p.Cys1055Ser and c.3625T>A:p.Ser1209Thr (Figure 4a,b), respectively). The first variant was described as pathogenic, according to the ClinVar database and the Mutation Taster program. The second variant presents a conflict of interpretations between being pathogenic and of uncertain significance, but according to the Mutation Taster program, this alteration can be harmful to the BLM protein. In addition, this change can generate a new splicing site and is in a highly conserved region.

### 2.2 Transcriptome using RNA‐seq methodology

The study of transcriptome based on RNA‐Seq methodology using the Illumina's HiSeq 2,500 platform (Illumina, Inc.) was performed as of approximately 2 ml of peripheral blood sample collected in PAXgene tube from two patients affected by BS (K1 and M1) and three unaffected controls (C1, C2, and C3).

RNA was isolated from QIAcube^®^ semi‐automated workstation QIAGEN^® ^using PAXgeneTM Blood RNA System Kit QIAGEN^®^ (Qiagen Sciences Inc., Germantown, MD, USA) following the manufactured protocol. After extraction, total RNA obtained was quantified by spectrophotometry at NanoDrop^®^ ND‐1000 (NanoDrop Thermo Technologies) and its integrity was determined by Agilent^®^ 2100 Bioanalyzer chip (Agilent Technologies), following the protocols described by the manufacturer. Samples were processed using TruSeq RNA sample prep protocol v2 LS Illumina^®^ protocol following the instructions described by the manufacturer for achievement of messengers RNAs (mRNA). Clusterization and sequencing was carried out in Illumina^®^ HiSeq 2500 platform (Illumina, Inc.).

Raw data obtained by Illumina^®^ HiSeq 2500 (Illumina, Inc.) were analyzed with Bowtie2 and EdgeR software with filtering reads for differential gene expression detection through four main steps: data filtering, mapping, differential expression analysis, and functional enrichment analysis of genes considered differentially expressed.

Filtering of low‐quality reads, adapters sequences and vectors was performed by the program Seqyclean (https://bitbucket.org/izhbannikov/seqyclean), using cutoff bases with quality less than 24 QScore. We also used Univec database (http://www.ncbi.nlm.nih.gov/VecScreen/UniVec.html). After filtering, reads with a length of less than 65 bp were removed.

Mapping of samples used reference genome of Homo sapiens (hg19, Genome Reference Consortium GRCh37 build, February 2009) by the Bowtie2 v2.1.0 (Langmead & Salzberg, 2012). Quality of mapping was checked for each sample using Samtools v.0.1.18 package/flagstat tool (Li et al., 2009).

Differential gene expression was performed using EdgeR tool. The biological conditions used for comparison were BS versus healthy controls that did not present the disease. In this step, as a guide,.gtf annotation file of reference genome previously used to extract the raw counts was given by the HTSeq‐count v.05.4.p2 script (http:// www huber.embl.de/users/anders/HTSeq/doc/index.html). Fold change (FC) coefficient determines the values for differential expression: Downregulated or Hipoexpression (<=−2) and Upregulated or Hyperexperssion (>=2); and pValue (*p* < .05).

Genes considered differentially expressed were submitted to a functional enrichment analysis based on gene ontology performed in the GO—GeneOntology Consortium (://www.geneontology.org) (Huang et al., 2009) and classified according the three categories enriched with p value (*p* > .05): Biological process, molecular function, and cellular components.

## 3 RESULTS

**Figure 5:**
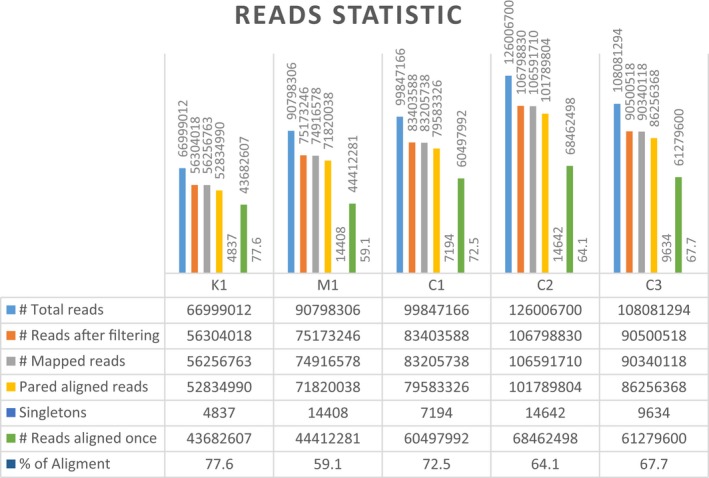
The libraries statistical results of Bloom´s syndrome group and controls

Five individuals (three healthy controls and two BS patients) were sequenced by the RNA‐seq methodology using Illumina HiSeq 2500 sequencing platform. Raw data generated by sequencing were processed, obtaining a highly complex transcriptional scenario, in which it was possible to observe the precise location of the transcription limits, with a single nucleotide resolution and high level of efficiency as shown in Figure 5 (Data S2: Supplementary RNA‐Seq Data Table Reads Statistic).

**Figure 6:**
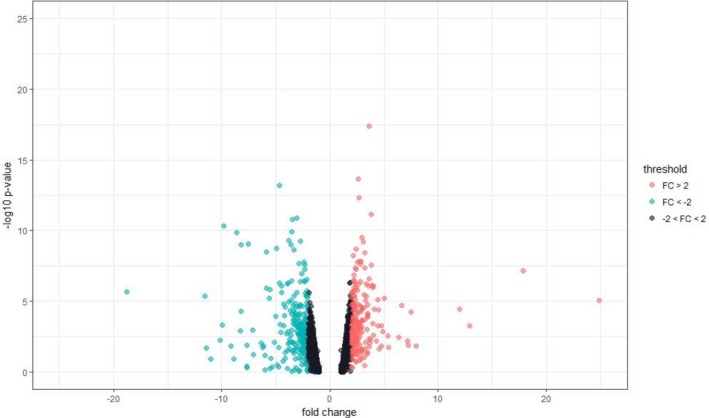
Volcano plot—The green dot shown upregulated differentially expressed genes; Gray dots represent genes that is not significant expression alteration and the red dots represent downregulated differentially expressed genes

**Figure 7:**
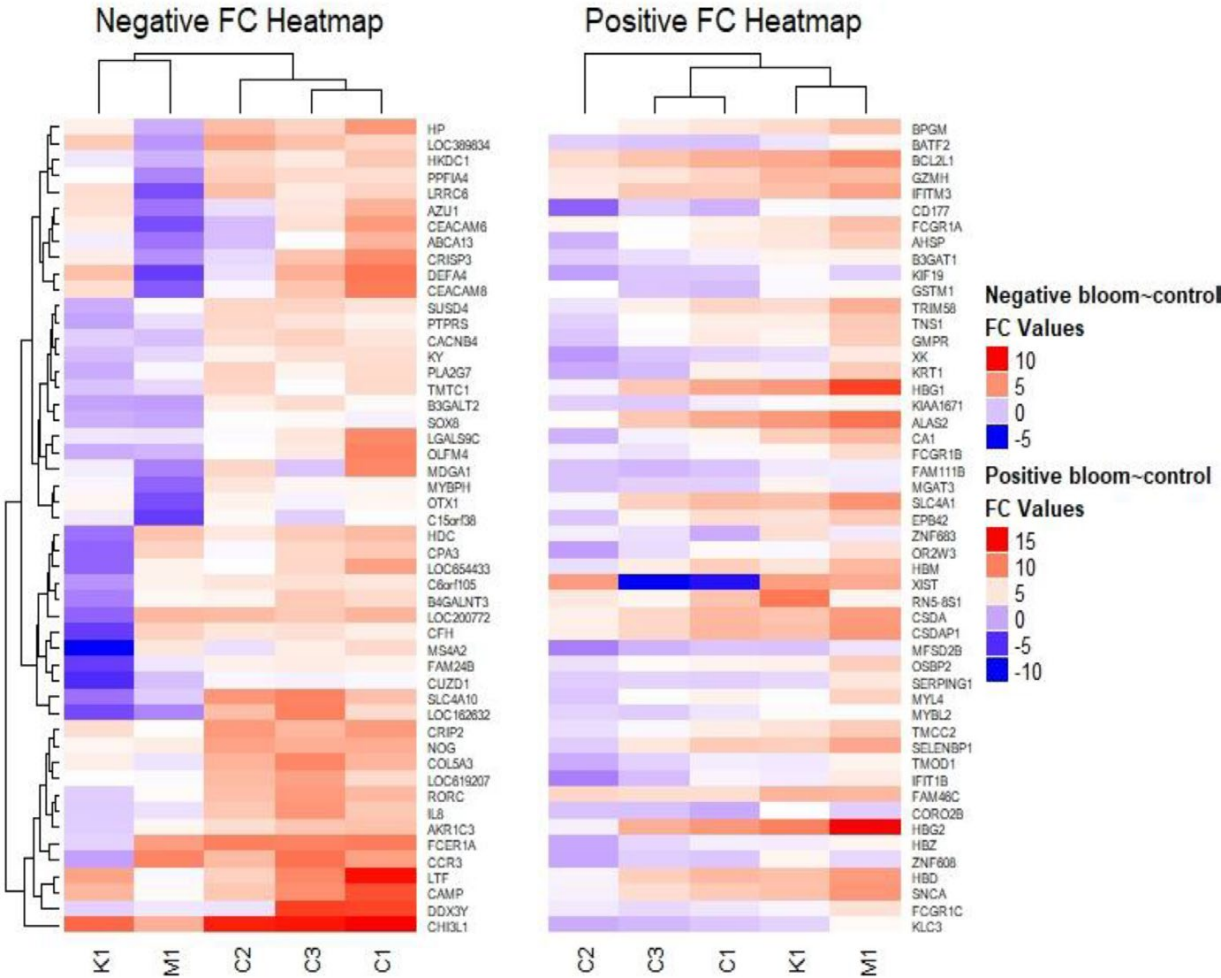
Heat map showing the top 50 profiles of significantly differentially expressed genes between Bloom´s syndrome group and health controls group

With this approach, 22.334 genes were mapped to the reference genome. Of these, a total of 11,938 presented the minimum coverage required for differential expression analysis (Data S2: Supplementary RNA‐Seq Data Table Sheet). Among these 11.938 genes, 492 were considered differentially expressed (DE) among BS patients and controls, as observed in Figure 6. Of these 492 genes, 282 were determined as downregulated (hypo‐expressed) and 210 upregulated (hyperexpressed) according Fold Change levels (Data S2: Supplementary RNA‐Seq Data Table Sheet). The significantly top 50 DE genes are shown in Figure 7.

**Figure 8:**
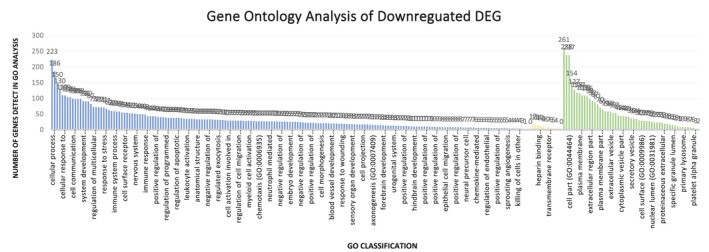
Statistical description of the gene ontology analysis of downregulated genes

**Figure 9:**
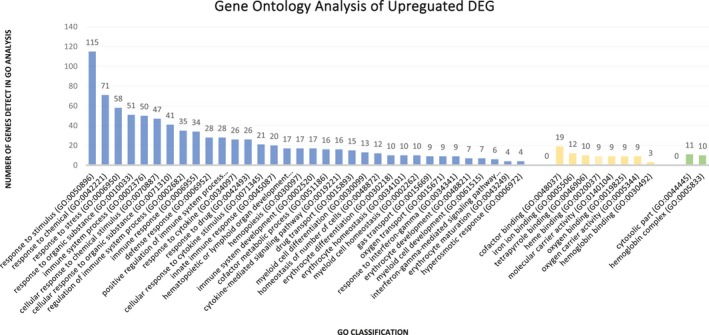
Statistical description of the gene ontology analysis of upregulated genes

All DE genes were submitted to functional enrichment analysis, and were classified according the three terms of gene ontology (GO). For the 282 downregulated DE genes, 254 were annotated into 247 process, being 184 genes related to biological process, 10 related to molecular functions, and 53 related to cellular components (Figure 8) (Data S3: Supplementary GO analysis). For the 210 upregulated DE genes, 198 were annotated into 44 process, being 34 genes related to biological process, 8 related to molecular functions, and 2 genes related to cellular components (Figure 9) (Data S3: Supplementary GO analysis).

The major processes related to genes that presented both downregulation and upregulation are associated with immunological pathways: negative regulation of viral genome replication, positive regulation of B cell proliferation, interferon gamma‐mediated signaling pathway, activation of B cell responses, virus response, adaptive immune response, and immune effector process.

Among these, the genes that present greater functional importance are those related to immune processes (GO: 0002376), genes related to the immune response (GO: 0006955), and genes related to the defense response (GO: 0006952). Some of the genes were important for more than one process, including *FCGR1B*,* KLRC2*,* CIITA*,* MS4A1,* and *FCGR2C*.

In addition, we also observed DE genes associated with apoptosis control such as apoptotic process in the bone marrow (GO: 0071839), apoptotic process (GO: 0006915), programmed cell death (GO: 0012501), and cell death (GO: 0008219). Some of these genes are *BCL2L1*,* CASP7*,* CDKN1A*,* E2F2*, *ITPR, CD274*, *TNFAIP6*, *TNFRSF25, TNFRSF13C,* and *TNFRSF*17.

Unexpectedly, no genes related to DNA repair pathways were detected, and the *BLM *gene, considered in the literature as responsible for BS, had normal levels of expression in relation to the control group in both patients by this methodology.

## 4 DISCUSSION

Our results indicate that genes associated with immune response and apoptosis control presented abnormal expression profile in two patients with BS. These abnormalities may contribute to the underlying pathophysiology of the disease. Nevertheless, genes related to DNA repair pathway showed similar expression to controls. This latter finding is contrary to what we originally thought, since BS is classically classified as a DNA repair deficiency syndrome.

A total of 216 differentially expressed genes were observed in the BS group. They were annotated to obtain the association with biologically relevant pathways associated with BS. The transcriptional profile of the 216 down‐ and upregulated (hypo‐expressed and hyperexpressed) DE genes found in the BS group in relation to the controls is mainly related to immunological pathways such as the recognition and antigen presentation, activation, and regulation of the complement system, among others.

Both patients presented typical clinical manifestations of BS, including microcephaly, growth deficiency, physical dysmorphisms, and clinically relevant immunodeficiency, characterized by recurrent infections. However, the immunological evaluation did not reveal any specific deficiency, such as number of innate/adaptive response cells, immunoglobulins production, or complement compromise. Indeed, several of the genes demonstrated to have abnormal expression are not associated with specific Mendelian immunodeficiencies in OMIM database, except for *MS4A1* (associated with autosomal recessive common variable immunodeficiency [OMIM: 613495], clinically manifested with recurrent bacterial respiratory infections, normal numbers of B cells, reduced numbers of memory B cells, B cells lacking surface CD20 expression, normal numbers of T cells, and defective antibody production, particularly T cell‐independent, hypogammaglobulinemia, low‐serum IgG and IgA, low‐ or normal‐serum IgM) and *CIITA* (associated with autosomal recessive bare lymphocyte syndrome [OMIM: 209920], clinically characterized by severe respiratory tract infections, malabsorptive diarrhea, neutropenia, severe autoimmune cytopenia, absence of humoral and cellular immune response to foreign antigens, normal number of T and B lymphocytes, reduced CD4+ count, and proportionally increased CD8+ count, panhypogammaglobulinemia or agammaglobulinemia). Although these two genes may contribute to immunological findings in BS especially if we consider their broad impact in several immune pathways, we believe that the sole contribution of dysregulated expression of these genes is not enough to explain the immunological manifestations of the patients, as both patients do not present the more severe clinical manifestations and laboratorial findings typically associated to *MS4A1‐* and *CIITA*‐related Mendelian disorders. The contribution of these two genes is part of a much more complex process.

Some BS patients, however, may present abnormal laboratory findings, such as reduction of serum immunoglobulin concentrations, decreased numbers of CD4‐positive T cells and impaired T cell proliferation in vitro, and impaired T helper cell function (Cunniff et al., 2017; Resende, Pereira, Melo, Santos, & Aguiar, 2007; Sanz, German, & Cunniff, 2016). Immune compromise in BS is classified in the group of combined immunodeficiencies (CID), according to the last classification consensus (Schoenaker et al., 2018). Common findings of CID groups are low number of memory T cells and increased naïve T cells. Nevertheless, a recent study observed subnormal levels of T, B, and NK cells and immunoglobulins in patients with BS and relatively mild infections, while their main laboratorial finding was a relative increase of effector memory T cells compared to naïve and central memory T cells (Schoenaker et al., 2018). This abnormal proportion of T cells is the opposite of the main CID conditions. Varying degrees of cellular and humoral immunodeficiencies of unknown etiology may also be observed. Cunnif et al. (2017) explained the abnormalities observed in both T and B cells to an impairment of the adaptive immune system as the source of the immune compromise seen in many affected individuals. We found additional pieces of evidence that corroborates the participation of dysregulated adaptive immune system: the transcriptional profile depicted altered expression profile involving *CD79A*, *FCGR1B*, *TNFRF13C*, *TNFRF17,* and *ICOSLG*, genes that participate in adaptive response. Among these genes, *CD79A* is associated with autosomal recessive agammaglobulinemia 3 [OMIM: 613501] that is clinically characterized by adult‐onset recurrent respiratory infections, decreased immunoglobulins, decreased number of mature B cells, and normal T cell numbers.

Another important finding implicates that defects in the innate response could also contribute to recurrent infections in BS patients, since another group of differentially expressed genes observed in our work includes *AZU1*, *BPI*, *CAMP*, *CTSG*, *DEFA4*, *LTF*, *PPBP*, *HP*, and *CXCL1*, which play role in regulation of innate immunity. None of these genes are associated with Mendelian conditions or monogenic forms of immunodeficiency in OMIM database. Therefore, our work found evidence of complex dysregulation involving both innate and adaptive immune system.

Autoimmune manifestations are not main findings described in BS. Indeed, both patients do not present clinical and laboratorial findings related to autoimmune diseases. A group of genes related to autoimmunity were demonstrated to be downregulated in our work. This group includes *ILR2RA* and *FOXP3*: the former participates in development, maintenance and activation of regulatory T cells (Tregs), while the latter is activated in the core of nucleus to promote activation of Tregs. Alterations in gene expression by Tregs can disturb the maintenance of immune homeostasis and failures in their development and function are the primary causes of a fatal autoimmune and inflammatory disease called immunodysregulation polyendocrinopathy enteropathy X‐linked (IPEX) syndrome (Goudy et al., 2013).

Several DE genes observed in our work such as *CD274*, *TNFAIP6*, *TNFRSF25, TNFRSF13C, and TNFRSF*17 were related to the activation and regulation of apoptosis in bone marrow and lymphocytes, a mechanism of programmed cell death. This process is generally characterized by morphological changes in the cell and activations of biochemical processes is considered a vital mechanism that involves several normal biological processes, such as cellular development, immune system functioning, and embryonic development, among others (Morimoto, Kaneko, Isogai, Kasahara, & Kondo, 2002). A cell that has dysregulated apoptosis mechanism will not suffer programmed death and thus may accumulate mutations and pass them on to the next cell generation. In addition, the response to apoptosis occurs in response to proteolysis of the BLM protein, thus binding to *BLM* gene expression (Wang & Hu, 2008). Literature also reported that apoptosis mechanisms are strongly related to BS, being responsible for short stature, as observed in our two patients (Kaneko, Fukao, Kasahara, Yamada, & Kondo, 2011).

Alternatively, it is possible that alteration of the regulatory mechanisms of apoptosis could generate a genomic instability, which affect the immune system and may be leading to neoplastic conditions, since the cells tend to accumulate damages (mutations) that can alter the cell cycle progression. Indeed, some anti‐ or pro‐apoptotic genes, as some members of BCL family, as *BCL2* and *BCL7A*, can indirectly be involved with neoplasias due to their capacity to activate caspases. BS patients are prone to the early development of multiple malignances of all types and at all locations (Chen, Xu, & Zhao, 2019; German, 1997), being the main cause of death in these patients (Amor‐Gueret, 2006; Ma, Corry, Rischin, Leong, & Peters, 2001). The main types of cancer include leukemia, lymphomas, breast, prostate, lung, and, in some cases,* Wilm's *tumor and retinoblastomas (Bhisitkul & Rizen, 2004; Chen et al., 2019; Cunniff et al., 2017; Ellis et al., 1998; German, 1997; Liu & West, 2008; Moreira et al., 2013). The propensity for developing cancer is probably liked to genomic instability. Our patients had not yet presented tumor manifestations, and this may be due to the detected hypoexpression of several tumorigenesis‐related genes such as *GLI1, KIT, WNT7A, AXIN2, FGFR1, FZD3, FZD6, ITGA6, PDGFB, PLEKHG5, and PRKC*.

Our study is limited to only two patients with BS. This restriction may have played important role in the fact that genes related to DNA repair pathway showed similar expression to controls. Therefore, larger groups of BS patients are still needed to better understand the expression profile, especially of DNA repair pathway genes, of patients with this genetic disorder. Nevertheless, we took precautions to minimize this limitation. For differential gene expression analysis, the use of biological replicates for the same group is recommended. This repetition does not necessarily contribute to the increase of the precision of the experiment, however, could be important for improving the accuracy of the means and other functions estimates of the response variables, contributing to increase the reliability of these estimates and the sensitivity of the experiment, to detect small but important differences in expression (Kvam, Liu, & Si, 2012). However, since the patients in this study had a rare disease, the analysis was performed in two patients affected by BS (patients BS1 and BS2) versus the control group (composed of three healthy individuals; C1, C2, and C3). Despite the restricted number of samples, we obtained extremely valuable results, which can explain the behavior of the disease.

Our results suggest that the combination of altered expression of genes involved in signaling pathways of immune response and apoptosis control may contribute directly to the main characteristics observed in BS, such as recurrent infections, growth failure, and high risk of cancer. Transcriptome studies of other instability syndromes could allow a more accurate analysis of the relevant gene interactions associated with the destabilization of the genome. This is a first description of the profile of differential gene expression related to immunological aspects detected in patients with BS by RNA‐seq.

## CONFLICT OF INTEREST

The authors declare that there is no conflict of interest regarding the publication of this paper.

## ETHICS APPROVAL AND CONSENT TO PARTICIPATE

This study was approved by the ethics committee of the University of São Paulo (HCFMUSP‐CAPPesq 63954/12) and the patients or parent of the patients signed the consent form for participation in the study.

## Data Availability

Seqyclean Program is available at ://bitbucket.org/izhbannikov/seqyclean. UniVec Database is available at ://www.ncbi.nlm.nih.gov/VecScreen/UniVec.html. HTSeq: Analyzing high‐throughput sequencing data with Python is available at http://www-huber.embl.de/users/anders/HTSeq/doc/index.htmlHTSeqcount v.05.4.p2 script. Database for Annotation, Visualization and Integrated Discovery (DAVID) is available at https://david.ncifcrf.gov. GeneOntology‐Consortium ://www.geneontology.org
